# Supplementary material for: Chirality-driven orbital magnetic moments as a new probe for topological magnetic structures
Source: Nat Commun. 2016 Dec 20;7:13613. doi: 10.1038/ncomms13613 (PMC5187428; doi:10.1038/ncomms13613)
Supplement: Supplementary Information — Supplementary Figures 1-3, Supplementary Notes 1-2 and Supplementary References. [file ncomms13613-s1.pdf]

SUPPLEMENTARY FIGURES

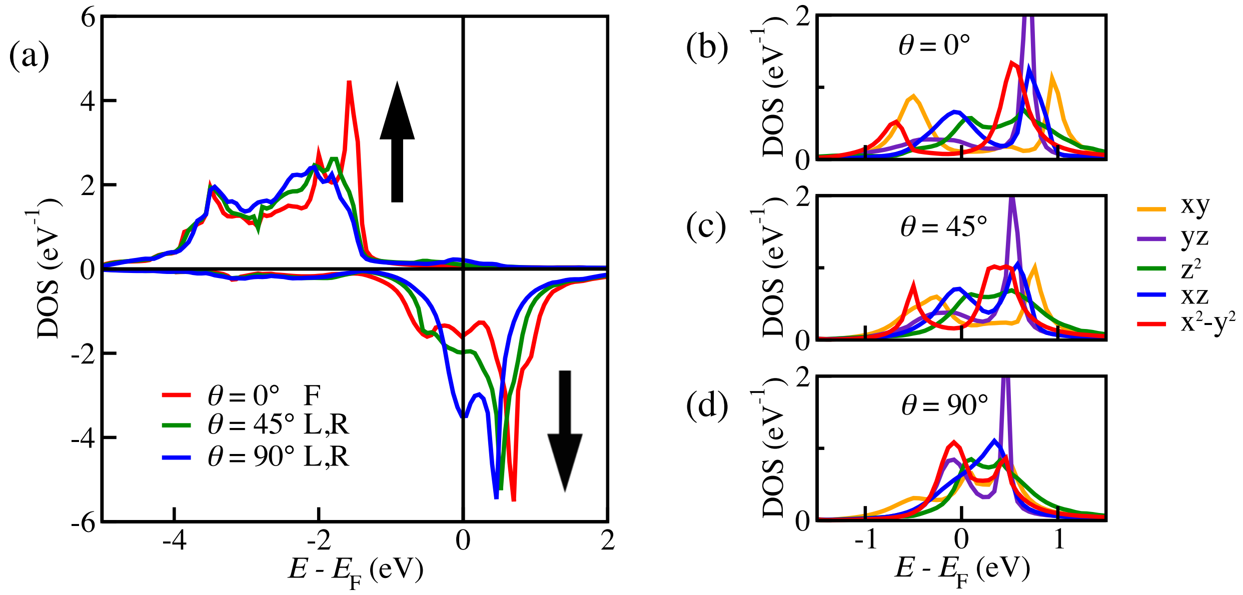

Supplementary Figure 1. **Electronic structure of Fe_3 on Cu(111).** (a) Spin-projected local density of states (DOS) with spin-orbit coupling, for Fe #1, in three different magnetic structures: ferromagnetic (F), chiral right-handed (R) and chiral left-handed (L). The arrows indicate majority (up) and minority (down) contributions, projected along the direction of the spin moment. θ is the polar angle between the spin moments and the surface normal, while different magnetic structures are specified by the azimuthal angle. When all three spin moments lie in the same plane ($\theta = 90°$) the L and R structures form a triangular Néel structure with $C_{123} = \mathbf{S}_1 \cdot (\mathbf{S}_2 \times \mathbf{S}_3) = 0$. (b–d) Orbital-resolved minority DOS for Fe #1, in the (b) $\theta = 0°$ (F); (c) $\theta = 45°$ (L,R); and (d) $\theta = 90°$ (L,R Néel) structures. The z-axis is the one normal to the surface, while the x-axis lies in the mirror plane passing through Fe #1 (the trimer has C_{3v} symmetry, but each magnetic atom has local C_s symmetry).

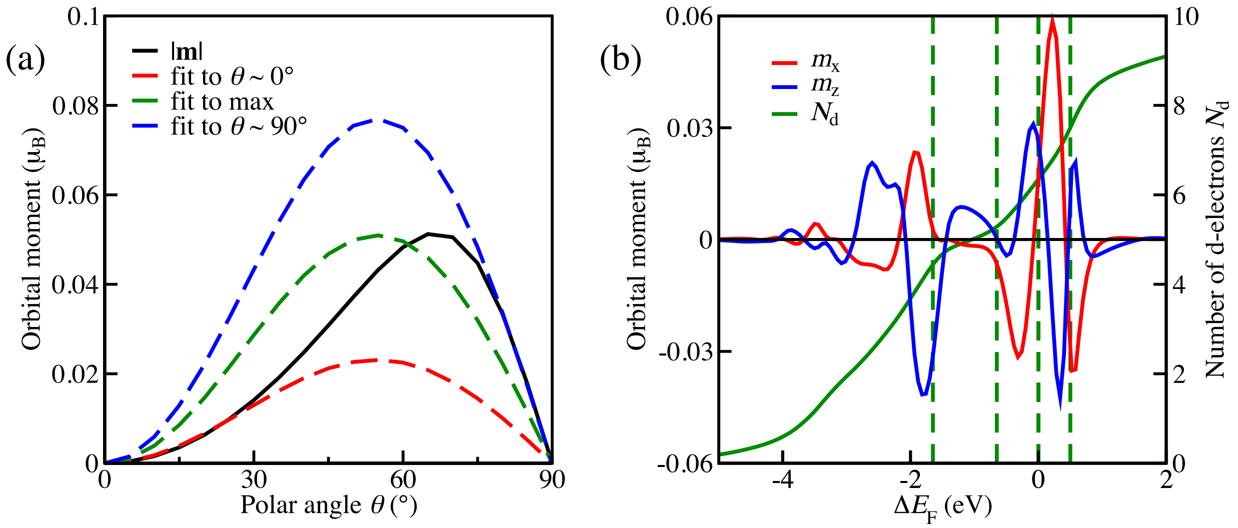

Supplementary Figure 2. **Chirality-driven orbital moments of Fe_3 on Cu(111).** (a) Orbital magnetic moment, $|\mathbf{m}|$, for Fe #1, without spin-orbit coupling, for the chiral right-handed (R) structure. The dashed lines show a comparison with the angular dependence of $C_{123}(\theta) = \mathbf{S}_1 \cdot (\mathbf{S}_2 \times \mathbf{S}_3) \propto \sin^2\theta\cos\theta$, with the amplitude chosen to match the data near $\theta = 0°$, $\theta = 90°$, or θ near the maximum value of the orbital moment. (b) Dependence of the two non-vanishing components, m_x and m_z, of the orbital moment of Fe #1 on the number of filled d-states (N_d), without spin-orbit coupling and for the R structure ($\theta = 45°$). We take the electronic structure of the Fe_3 cluster as reference, and vary the Fermi energy with respect to the true one (ΔE_F). This reveals the dependence of the orbital moment on which d-states are filled. From left to right, the vertical dashed lines mark the fillings corresponding to Cr, Mn, Fe and Co, respectively.

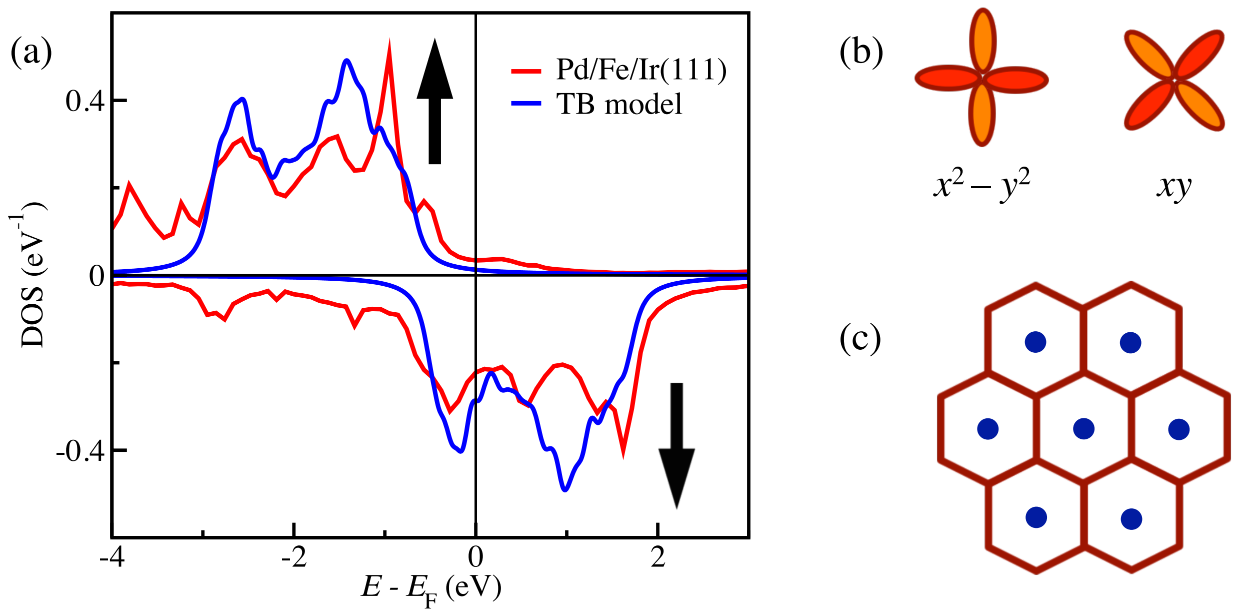

Supplementary Figure 3. **Electronic structure of Fe layer in Pd/Fe/Ir(111).** (a) Spin-projected local density of states from density functional theory calculations [1] and from the tight-binding model, Supplementary Equation 1. Only the contribution of x^2-y^2 and xy character is shown. The arrows indicate majority (up) and minority (down) projections. (b) The two orbitals selected for the construction of the tight-binding model, viewed along the z-axis (normal to the Fe atomic plane). (c) Sketch of the hexagonal skyrmion lattice obtained by applying periodic boundary conditions to the hexagonal unit cell. The red hexagons mark the boundary of the unit cells, where the spins always point up, while the blue circles represent the skyrmion cores, where the spins point antiparallel to those in the boundary.

SUPPLEMENTARY NOTES

Supplementary Note 1 — Electronic structure calculations for trimers

The electronic structure calculations for the trimers are performed within density functional theory (DFT), as implemented in the Korringa-Kohn-Rostoker Green function method [2], using the atomic sphere approximation with full charge density, a cutoff of $\ell_{\mathrm{max}} = 3$, and the local spin density approximation parametrized by Vosko, Wilk and Nusair [3]. First a self-consistent calculation is performed for a slab of 22 Cu layers stacked in the fcc (111) direction, terminated with two vacuum regions equivalent to three more atomic layers on each surface. Then a real-space cluster is self-consistently embedded on the Cu(111) surface, consisting of the magnetic trimer and nearest-neighbor atoms. Different magnetic structures can be stabilized employing constraining fields [4, 5], and are illustrated in Figure 1 of the main text.

The local density of states (DOS) with spin-orbit coupling (SOC) is shown in Supplementary Figure 1(a), for three magnetic structures of Fe_3. The largest energy scale is provided by the exchange splitting, leading to well-separate majority and minority states, even when SOC is included in the calculation. The peak splittings in the minority DOS decrease with increasing polar angle θ, as the noncollinearity reduces the effective hybridization between magnetic sites. Supplementary Figure 1(b–d) shows the orbital-projected DOS for the same magnetic structures. Although the trimer has $C_{3\mathrm{v}}$ symmetry about its center, each atom has only C_{s} symmetry (one mirror plane). The character of the d-states near the Fermi energy determines the properties of the orbital magnetic moments — orientation and magnitude. The orbital moments are computed using the atomic orbital angular momentum operator, for each magnetic site.

Supplementary Figure 2(a) compares the angular dependence of the chirality-driven orbital moment for Fe #1 in the R structure with the angular dependence of the scalar spin chirality, $C_{123}(\theta) = \mathbf{S}_1 \cdot (\mathbf{S}_2 \times \mathbf{S}_3) \propto \sin^2\theta\cos\theta$. The shape of the curves roughly agree, but the maxima are located at different angles θ_{max}. The angular function $C_{123}(\theta)$ can still be used for a range of angles near either the ferromagnetic or the triangular Néel states. The explanation for the discrepancies is given in Supplementary Figure 1(b–d): as the magnetic structure changes with the polar angle, the orbital occupations and splittings near the

Fermi energy change too, leading to a more complicated angular dependence of the orbital moment.

Supplementary Figure 2(b) addresses the dependence of the chirality-driven orbital moment on the filling of the d-states, keeping the magnetic structure fixed (R, $\theta = 45°$). As for the SOC-driven orbital moment, the chiral orbital moment has largest magnitude when the d-states are partially filled (compare with Supplementary Figure 1(a)). The dashed lines mark the fillings corresponding to the other magnetic species; there is good agreement with the data for the other magnetic trimers shown in Figure 2(b–d) of the main text.

Supplementary Note 2 — Electronic structure calculations for skyrmions

The minimal tight-binding model for the skyrmionic structures is constructed using the DOS from ferromagnetic DFT calculations for Pd/Fe/Ir(111), see Supplementary Figure 3(a). The magnetic Fe layer forms a hexagonal lattice with the lattice constant of the Ir(111) substrate. We select two degenerate d-orbitals, $|x^2-y^2\rangle$ and $|xy\rangle$, see Supplementary Figure 3(b), as the largest contribution to the orbital moment comes from degenerate orbitals that can be remixed by SOC or by the emergent magnetic field. The Hamiltonian consists of local exchange coupling to prescribed spin directions ($\mathbf{n}_i$) and hopping to nearest-neighbors only, $\langle i,j\rangle$:

$$\mathcal{H}_{\mathrm{TB}} = -J \sum_i \sum_{mss'} c^\dagger_{ims}\, \mathbf{n}_i \cdot \boldsymbol{\sigma}_{ss'}\, c_{ims'} + \sum_{\langle i,j\rangle} \sum_{mm's} c^\dagger_{ims}\, t_{im,jm'}\, c_{jm's} \quad . \tag{1}$$

Here i labels the sites, m the orbitals and s the spins, with $\boldsymbol{\sigma}$ the vector of Pauli matrices.

Taking into account the shape of the orbitals, the hopping matrix is represented by

t_{ij}	$\|x^2-y^2\rangle$	$\|xy\rangle$
$\langle x^2-y^2\|$	$t\left(1+\cos 4\phi_{ij}\right)$	$t\sin 4\phi_{ij}$
$\langle xy\|$	$t\sin 4\phi_{ij}$	$t\left(1-\cos 4\phi_{ij}\right)$

, (2)

with the angle ϕ_{ij} between the direction of the ij-bond and the x-axis. We take $J = 1.2$ eV, $t = 0.5$ eV, include SOC on each site via $\mathcal{H}_{\mathrm{SOC}} = \xi \sum_i \mathbf{L}_i \cdot \mathbf{S}_i$ with $\xi = 0.025$ eV (here $\mathbf{L}_i$ and $\mathbf{S}_i$ are the orbital and spin angular momentum operators for each site), mimic the effect of

hybridization with all other neglected electronic states by a constant broadening $\mathit{\Gamma} = 0.1$ eV, and set the band filling to $N_d = 2.5$. In the ferromagnetic state the spin moment per atom is 1.4 μ_{B}, and the SOC leads to an orbital moment per atom of 0.1 μ_{B}. The corresponding electronic structure is shown in Supplementary Figure 3(a), where it is compared with the corresponding partial DOS from the DFT calculation.

To compute the electronic structure of skyrmionic magnetic structures, the remaining ingredient is the skyrmion profile, which controls the local exchange coupling to the directions $\mathbf{n}_i$. We adopt the radial profile, $\theta(r;B)$, from Ref. [6],

$$\theta(r;B) = \pi + \arcsin\left(\tanh\frac{r+c(B)}{w(B)}\right) + \arcsin\left(\tanh\frac{r-c(B)}{w(B)}\right) \tag{3}$$

which is a sum of two domain walls centered at $\pm c$ with width w; these parameters depend on the magnetic field B, as can be seen in Figure 2(e) of Ref. [6]. We employ the following parametrization based on the data shown in that figure:

$$c(B) = \frac{c_0}{1+B/B_0} \quad , \quad c_0 = 12 \text{ nm} \quad , \quad B_0 = 0.2 \text{ T} \quad ; \tag{4}$$

$$w(B) = w_0 - w_1\, B \quad , \quad w_0 = 0.8 \text{ nm} \quad , \quad w_1 = 0.08 \text{ nm T}^{-1} \quad . \tag{5}$$

The local spin direction is $\mathbf{n}_i = \big(\cos(m\phi_i)\sin\theta(r_i)\,,\,\sin(m\phi_i)\sin\theta(r_i)\,,\,\cos\theta(r_i)\big)$, with $r_i = 0$ the center of the skyrmionic structure, m the vorticity (how many times the spins wind around the center), and (r_i,ϕ_i) the polar coordinates of the sites in the hexagonal lattice, with $a_{2\mathrm{D}} = 2.72$ Å the lattice constant of the Ir(111) surface. The unit cell contains $31 \times 31 = 961$ sites, corresponding to a linear dimension of 31×0.272 nm $= 8.4$ nm; see Figure 3(a) of the main text for an illustration. Periodic boundary conditions lead to a skyrmion lattice, sketched in Supplementary Figure 3(c).

SUPPLEMENTARY REFERENCES

[1] D. M. Crum, M. Bouhassoune, J. Bouaziz, B. Schweflinghaus, S. Blügel, and S. Lounis, "Perpendicular reading of single confined magnetic skyrmions," Nat. Commun. **6**, 8541 (2015).

[2] N. Papanikolaou, R. Zeller, and P. H. Dederichs, "Conceptual improvements of the KKR method," J. Phys.: Condens. Matter **14**, 2799–2823 (2002).

[3] S. H. Vosko, L. Wilk, and M. Nusair, "Accurate spin-dependent electron liquid correlation energies for local spin density calculations: a critical analysis," Can. J. Phys. **58**, 1200–1211 (1980).

[4] P. H. Dederichs, S. Blügel, R. Zeller, and H. Akai, "Ground states of constrained systems: Application to cerium impurities," Phys. Rev. Lett. **53**, 2512–2515 (1984).

[5] B. Ujfalussy *et al.*, "Constrained density functional theory for first principles spin dynamics," J. Appl. Phys. **85**, 4824–4826 (1999).

[6] N. Romming, A. Kubetzka, C. Hanneken, K. von Bergmann, and R. Wiesendanger, "Field-dependent size and shape of single magnetic skyrmions," Phys. Rev. Lett. **114**, 177203 (2015).
